# Long-Term Survival with Daratumumab, Lenalidomide and Dexamethasone in Transplant-Ineligible Newly Diagnosed Multiple Myeloma Patients—A Survey from Two Italian Centers

**DOI:** 10.3390/diseases14020081

**Published:** 2026-02-21

**Authors:** Vittorio Del Fabro, Lara Gullo, Giuliana Giunta, Giuseppina Uccello, Claudia Bellofiore, Cristina Lo Faro, Dario Leotta, Federica Elia, Veronica Vecchio, Chiara Sorbello, Ugo Consoli, Alessandra Romano, Francesco Di Raimondo, Manlio Fazio, Fabio Stagno, Concetta Conticello

**Affiliations:** 1 Faculty of Medicine and Surgery, “Kore” University of Enna, 94100 Enna, Italy; 2 Hematology with BMT Unit, A.O.U. Policlinico “G. Rodolico-San Marco”, 95123 Catania, Italy; lara.gullo@virgilio.it (L.G.); dario.leotta96@gmail.com (D.L.); vero.vecchio99@gmail.com (V.V.); diraimon@unict.it (F.D.R.); ettaconticello@gmail.com (C.C.); 3 Division of Hematology, Azienda Ospedaliera di Rilievo Nazionale e di Alta Specializzazione Garibaldi, 95122 Catania, Italy; 4 Dipartimento di Specialità Medico-Chirurgiche, Dipartimento di Chirurgia Generale e Specialità Medico-Chirurgiche, Sezione di Ematologia, Università degli Studi di Catania, 95131 Catania, Italy; 5 Post-Graduation School of Hematology, University of Catania, A.O.U. “Policlinico-San Marco”, 95123 Catania, Italy; 6 Hematology Section, University of Messina, A.O.U. “Policlinico G. Martino”, 98124 Messina, Italy

**Keywords:** multiple myeloma, daratumumab lenalidomide dexamethasone, first line

## Abstract

Background: Multiple myeloma (MM) is a clonal plasma cell neoplasm representing the second most common hematological malignancy. The combination of daratumumab, lenalidomide and dexamethasone (D-Rd) was first approved by the EMA (European Medicines Agency) for the treatment of relapsed/refractory multiple myeloma (RRMM) patients, and was subsequently approved for first-line therapy, based on the results of POLLUX and MAIA trials, respectively. Methods: In this survey, we retrospectively collected data from 96 consecutive transplant-ineligible newly diagnosed multiple myeloma (TIE-NDMM) patients treated with the D-Rd combination. Results: The median age was 73 years; the median progression free survival (mPFS) and median overall survival (mOS) were not reached (NR); the overall response rate (ORR), defined as patients who obtained at least a partial response (PR), was 90%; 59% of patients achieved a very good partial response (VGPR) or better. A strong negative correlation was observed between treatment response and elevated beta-2-microglobulin levels. Conclusions: This study confirms the efficacy of the D-Rd combination as first-line therapy for TIE-NDMM patients, suggesting that achieving at least a PR—and particularly a VGPR—may represent a strong predictor of long-term remission and survival, even in the era of new combinations based on the use of quadruplets.

## 1. Introduction

Multiple myeloma is a hematologic malignancy characterized by a clonal plasma-cell proliferation leading to complications and, ultimately, death. Although recent therapeutic advances have significantly improved patient outcomes, multiple myeloma remains an incurable disease. It particularly affects the elderly, who often have frailty or comorbidities that limit their eligibility for intensive therapies, including autologous stem cell transplantation (ASCT) [1,2]. Initial treatment depends on the patient’s ability to tolerate the toxic effects of high-dose chemotherapy and autologous stem-cell transplantation [3]. For TIE-NDMM patients, typically defined by age, frailty status or organ dysfunction, the primary therapeutic goal is to achieve deep and durable responses using well-tolerated regimens. Multidrug regimens including glucocorticoids, immunomodulatory drugs and proteasome inhibitors have represented the standard of care [4,5,6,7]. In this context, the introduction of the anti-CD38 monoclonal antibody daratumumab has profoundly changed frontline treatment strategies [8,9,10,11], leading to good responses also in TIE-NDMM patients [12,13].

The D-Rd regimen emerged as a new standard of care following the results of the phase 3 multicenter MAIA trial, which demonstrated significant improvements in progression-free survival (PFS), overall survival (OS), and minimal residual disease (MRD) negativity compared with lenalidomide and dexamethasone (Rd) alone in TIE-NDMM patients [14]. In the updated analysis of the MAIA trial, with a median follow-up of 64.5 months, D-Rd maintained a substantial progression-free survival (PFS) benefit, reducing the risk of disease progression by approximately 45% [15,16,17].

A recent meta-analysis showed that D-Rd, compared with D-VMP (daratumumab, bortezomib, melphalan and prednisone) and VRd (bortezomib, lenalidomide and dexamethasone), had the highest chance of being ranked as the most effective treatment with respect to both PFS and OS [18,19,20,21,22].

More recently, quadruplet combinations, including anti-CD38 such as D-VRd (Daratumumab, Bortezomib, Lenalidomide, and Dexamethasone) and Isa-VRd (Isatuximab, Bortezomib, Lenalidomide, and Dexamethasone), have demonstrated promising efficacy in newly diagnosed MM patients [23,24,25,26,27,28]. The phase III IMROZ trial evaluated the quadruplet combination Isa-VRd in TIE-NDMM patients showing that the addition of isatuximab significantly reduced the risk of progression or death and increased complete response and MRD-negativity rates [29]. Recently, the EHA-EMN guidelines provided indications for use of quadruplets in first-line treatment for TIE-NDMM patients [30].

In this retrospective survey, we evaluated 96 consecutive TIE-NDMM patients treated with the D-Rd regimen at two Italian centers between March 2021 and March 2025. The aim of this study is to support the efficacy and safety of D-Rd association in TIE-NDMM patients in the real-world setting, confirming the efficacy and utility of this treatment even in this new era of therapies, with particular attention to frail and elderly patients. Given the observational and retrospective nature of the analysis, any comparison with clinical trial populations should be considered hypothesis-generating.

## 2. Materials and Methods

We evaluated 96 patients aged ≥70 or <70 with comorbidities that contraindicated high-dose chemotherapy for bone marrow transplantation. The diagnosis was made using the International Myeloma Working Group (IMWG) criteria, as well as the response criteria [31,32,33]. Only patients who received at least one administration of each study drug were included in the analysis, with a predefined cut-off date of 31 March 2025. All participants provided written informed consent in accordance with the Declaration of Helsinki and the study was approved by the ethics committees of the participating institutions.

Daratumumab was given subcutaneously and no dose reduction was performed, according to the approved schedule; only treatment delays were allowed when clinically indicated. Dexamethasone was administered orally at a dose of 20 mg once weekly and was reduced in cases of treatment-related toxicity or intolerance. Lenalidomide was administered at doses ranging from 5 to 25 mg daily on days 1–21 of each 28-day cycle, according to the manufacturer’s guidelines. Dose adjustments were made based on clinical conditions, renal function, and the occurrence of cytopenias. Concomitant medications included thromboprophylaxis with low-dose aspirin for low-risk patients and low-molecular-weight heparin or oral anticoagulants for high-risk patients. Anti-infective prophylaxis consisted of trimethoprim–sulfamethoxazole 800 mg twice daily on two days per week and acyclovir 200 mg once daily. All patients were candidates for annual influenza and pneumococcal vaccination. Almost all patients received vaccination against SARS-CoV-2 in 2021 (at least two doses), and in the following year, tixagevimab–cilgavimab was administered as pre-exposure prophylaxis against SARS-CoV-2 infection, in accordance with therapeutic indications approved by the European Medicines Agency and the Italian Medicines Agency [34,35]. Baseline clinical and disease characteristics are reported in Table 1. Frailty status was assessed using the simplified frailty score proposed by Facon et al. [36], which stratifies patients according to age, Charlson Comorbidity Index (CCI), and Eastern Cooperative Oncology Group (ECOG) performance status, classifying them as frail or non-frail. The presence of para-osseous and/or extramedullary disease was evaluated using computed tomography (CT) and/or magnetic resonance imaging (MRI), in association with fluorodeoxyglucose positron emission tomography (FDG-PET) [37,38,39]. Cytogenetic risk was defined based on the presence of del17, gain/amp1q, t(4;14), t(14;16) and t(16;20); patients with one cytogenetic abnormality were classified as high risk, whereas those with two or more abnormalities were classified as very high risk [40,41]. Adverse events (AEs) were evaluated according to the Common Terminology Criteria for Adverse Events v5.0 (CTCAE v5.0). Treatment responses were assessed according to the International Myeloma Working Group (IMWG) response criteria [31].

## 3. Results

### 3.1. Patients’ Characteristics

The 96 analyzed patients were equally distributed by sex (48 females and 48 males). The median follow-up was 23 months (range, 2–53), and the median age was 73 years; 32 patients were aged ≥ 75 years and 23 were aged ≤ 70 years; overall, 50 patients (52%) had an ECOG performance status ≥ 2, and 75 patients (78%) were classified as frail. Fluorescence in situ hybridization (FISH) analysis was performed in most patients; however, results were available in only 44 cases due to technical failure of the analysis, with 5 patients classified as having very high cytogenetic risk. Eight patients presented with para-osseous involvement at diagnosis, and eight had extramedullary disease (EMD). Fifty-nine patients had beta-2-microglobulin levels above 3.5 mg/L, and 22 patients showed elevated lactate dehydrogenase (LDH) levels. According to the International Staging System (ISS), 48% and 24% of patients were classified as stage III and stage II, respectively. The most frequently used starting dose of lenalidomide was 10 mg (40 patients), followed by 15 mg and 25 mg (24 and 25 patients, respectively), based on comorbidities and renal function, in accordance with the manufacturer’s recommendations. Only seven patients started at a dose of 5 mg due to renal impairment. Dose reductions of lenalidomide were required in cases of grade 3–4 toxicity or persistent grade 2 toxicity (Table 1).

### 3.2. Statistical Analysis

Descriptive statistics were generated for data analysis and two sides *p*-values of 0.05 or less were considered significant. Qualitative results were summarized in counts and percentages. Continuous variables were summarized with the use of descriptive statistics, and categorical variables were summarized as numbers and percentages. The Overall Response Rate (ORR) was defined as achieving a PR or better. Time-to-event variables were estimated with the use of the Kaplan–Meier method and compared by the log-rank test. Univariate analyses were performed to evaluate the impact of age (≤70 vs. >70 years), sex, cytogenetic risk (standard, high, very high), ISS stage at diagnosis, presence of bone lesions, para-osseous or extramedullary disease, elevated LDH or beta-2-microglobulin levels, initial lenalidomide dose, lenalidomide dose reduction, and best response achieved. Fisher’s exact test was used for categorical variables with two categories; the χ^2^ test was for nominal variables with more than 2 categories. The Kaplan–Meier test analyzed PFS and OS. Standard errors were calculated by the method of Greenwood. PFS was defined as the time from treatment initiation to disease progression, relapse-related death, or last follow-up (31 March 2025). OS was defined as the time from treatment initiation to death from any cause or last known follow-up. Variables showing a significant correlation in Kaplan–Meier in Kaplan–Meier analyses were further evaluated using univariate and multivariate analyses. Multivariate analyses were performed using logistic regression models. The level of statistical significance was set at the 95th percentile. All calculations were performed using SPSS Statistics version 29.0.2.

### 3.3. Efficacy

**Figure 1:**
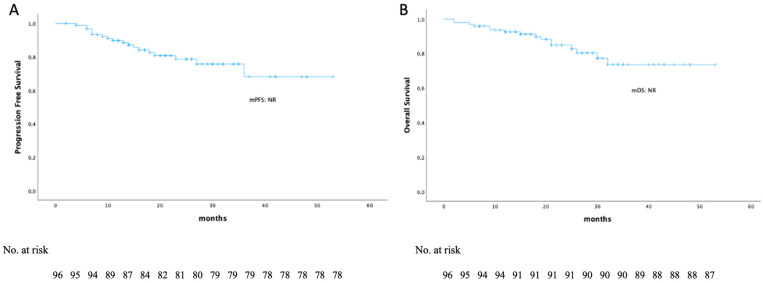
(**A**) Progression free survival in the entire patient population; the median PFS is not reached; (**B**) Overall survival in the entire patient population; the median OS is not reached.

After a median follow-up of 23 months (range, 2–53), the median PFS (mPFS) and median OS (mOS) were not reached (Figure 1). The median time to first response was 1 month (range 1–7) and the median time to best response was 6 months (range 1–32). The median OS (mOS) for patients who relapsed during treatment was 26 months, whereas it was not reached (NR) for non-relapsed patients (*p* < 0.001).

**Figure 2:**
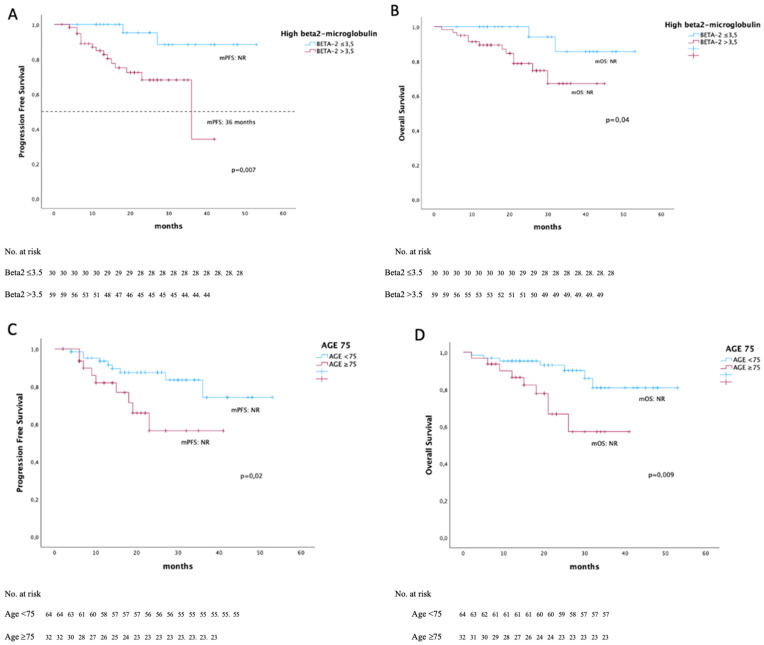
Kaplan–Meier curves based on beta2-microglobuline and age. (**A**) PFS stratified by beta2-microglobulin levels above or below 3,5 mg/L, with a significant difference between the two groups (*p* = 0.007). (**B**) OS stratified by beta2-microglobulin levels above or below 3,5 mg/L, with a significant difference between the two groups (*p* = 0.04). (**C**) PFS stratified by age < 75 years or older, with a significant difference between the two groups (*p* = 0.02). (**D**) OS stratified by age < 75 years or older, with a significant difference between the two groups (*p* = 0.02).

**Figure 3:**
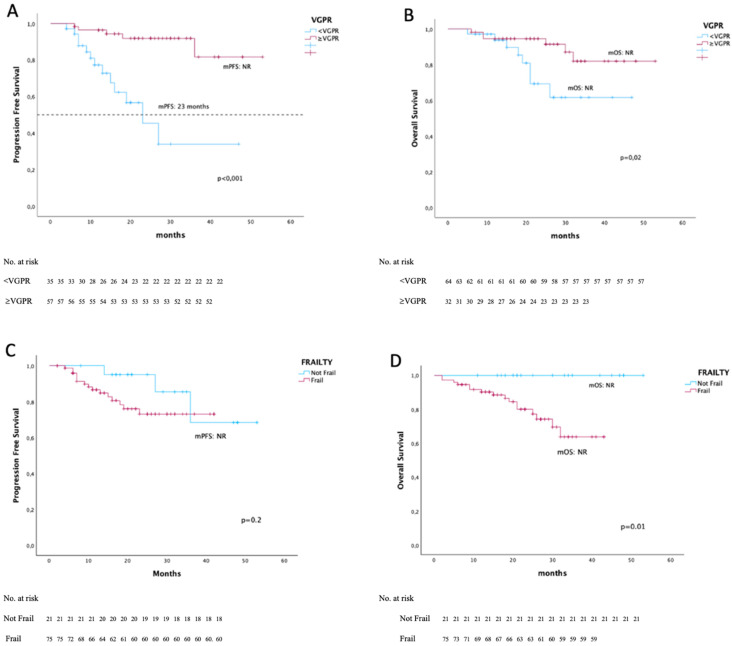
PFS and OS in the entire patient population based on whether or not a VGPR was achieved and age. (**A**) PFS and (**B**) OS stratified by whether or not at least a VGPR was achieved, with a significant difference between the two groups. (**C**) PFS and (**D**) OS stratified by frailty, with a significant difference between the two groups.

No significant difference in PFS was observed between patients aged <75 years and those aged ≥75 years (*p* = 0.2) (Figure 2). Conversely, frail patients showed a significantly worse OS compared with non-frail patients, although the median OS was not reached in either group (*p* = 0.01); no difference in PFS was observed between these groups (Figure 3). Frail patients showed a significantly worse OS compared with non-frail patients, although the median OS was not reached in either group (*p* = 0.01). Among the seven patients with del(17p), median PFS was 15 months compared with not reached in patients without this alteration (*p* < 0.001), while no significant difference in OS was observed (*p* = 0.3). Although a high proportion of patients were classified as ISS stage III (65%), no significant differences in PFS were observed according to ISS stage (*p* = 0.1). No association was found between PFS and the presence of para-osseous or extramedullary disease (*p* > 0.1), nor with elevated LDH levels (*p* = 0.3). In contrast, elevated beta-2-microglobulin levels were significantly associated with worse outcomes. Patients with normal beta-2-microglobulin values showed significantly longer PFS and OS compared with those with elevated levels (*p* = 0.007 and *p* = 0.04, respectively) (Figure 2) and these findings were confirmed in both univariate and multivariate analyses.

Lenalidomide dose reduction or treatment delays did not significantly affect PFS or OS (*p* > 0.1). Overall, 57.3% of patients required a reduction in the initial lenalidomide dose, most commonly due to gastrointestinal toxicity. The ORR was 90%, with 86 patients achieving at least a PR. Fifty-seven patients (59%) achieved a VGPR or better, including 26 patients (27%) who achieved complete response. Patients achieving at least a PR had significantly longer PFS compared with those who did not (median PFS not reached vs. 9 months, *p* = 0.006). This difference was even more pronounced when comparing patients achieving ≥VGPR versus <VGPR (median PFS not reached vs. 23 months) (*p* < 0.001) (Figure 3). Multivariate analysis confirmed that deeper responses, particularly achievement of ≥VGPR, were strong predictors of prolonged PFS (*p* < 0.001).

The general mOS was not reached. It was influenced by age (<75 or ≥75 years) (*p* = 0.009) with median NR for both groups, by the presence of paravertebral tissue (25 months vs. NR, *p* = 0.001) and high beta2-microglobulin values (*p* = 0.04) with median NR for both groups. OS was not influenced by cytogenetic risk (*p* = 0.1), ISS stage (*p* = 0.09), the presence of EMD (*p* = 0.1), elevated LDH (*p* = 0.4), and reduction in lenalidomide dose (*p* > 0.1). Patients who reached at least a VGPR had a better OS (mOS NR in both groups, *p* = 0.02).

### 3.4. Safety

Eighteen patients (19%) experienced disease progression and 16 patients (17%) died due to worsening clinical conditions and/or disease relapse. The most relevant (≥10%) grade 3–4 adverse events (AEs) are reported in Table 2 and were mainly gastrointestinal and hematological. Gastrointestinal toxicity, particularly diarrhea, was the most frequently observed AE, occurring in 18 patients (19%). Neutropenia was reported in 17 patients (18%), infections in 9 patients (15.3%) and anemia in 13 patients (14%) (Table 2). Diarrhea was most often grade 1–2 but was frequently persistent. It was managed with antidiarrheal agents and drugs reducing intestinal peristalsis, leading to partial or complete symptom improvement. In cases of persistent symptoms despite supportive therapy, lenalidomide dose reduction was required. Overall, lenalidomide dose reduction was performed in 55 patients (57%) due to treatment-related adverse events, with gastrointestinal toxicity accounting for 42% of these reductions. Importantly, lenalidomide dose reduction did not negatively impact progression-free or overall survival. Grade 3–4 infections, mainly pneumonia, represented the primary cause of treatment delays. Twenty-five patients (26%) experienced at least one treatment-cycle delay, 56% of which were attributable to infectious complications. In cases of severe infections, corticosteroid dose reduction was also applied. In patients experiencing neutropenia with neutrophil counts <1000/mm^3^ or anemia with hemoglobin levels <10 g/dL, hematopoietic growth factors were administered to reduce the risk of severe cytopenias or infectious complications.

Subgroup analysis according to age (<75 vs. ≥75 years) showed no significant differences in the overall incidence of adverse events between the two groups, with the exception of anemia. Grade 3–4 anemia occurred in 5 patients (8%) in the <75-year group and in 8 patients (25%) in the ≥75-year group (*p* = 0.02). However, this difference did not translate into significant differences in progression-free or overall survival in multivariate analysis.

## 4. Discussion

The addition of the monoclonal antibody daratumumab to the standard of care for multiple myeloma has led to substantial improvements in progression-free and overall survival, as demonstrated in several clinical trials, both in the frontline and relapsed/refractory settings.

The median age of patients in our cohort was identical to that of the MAIA trial (73 years); however, a higher proportion of patients were classified as ISS stage III compared with the MAIA population (48% vs. 29.1%). This finding may suggest a trend toward a more aggressive disease profile in our real-world cohort, although direct comparisons with clinical trial populations should be interpreted with caution. Despite this, treatment efficacy was not negatively influenced by ISS stage, supporting the effectiveness of D-Rd even in patients with more advanced disease. Response to treatment was rapid, with a median time to first response of 1 month (range 1–7) and a median time to best response of 6 months (range 1–32). These findings confirm the ability of the D-Rd regimen to induce early and deep responses, even in an elderly and frail population. Importantly, median PFS and OS were not reached in the overall population, underscoring the durability of clinical benefit achieved with this regimen. In both patients younger than 75 years and those aged 75 years or older, median PFS and OS were not reached. Nevertheless, statistically significant differences were observed between the two age groups for both PFS and OS, with inferior outcomes in older patients (*p* = 0.02 and *p* = 0.009, respectively) (Figure 2). These results highlight age as a relevant prognostic factor, even in the context of highly effective and well-tolerated regimens. The presence of del(17p) was associated with significantly shorter PFS (*p* < 0.001) but did not significantly affect OS. This observation seems to confirm the negative prognostic impact of del(17p), but we must always underline that these data are based on a limited number of patients. Similarly, cytogenetic risk was significantly associated with PFS, with worse outcomes observed in patients with high-risk features. The lack of a statistically significant difference in OS across cytogenetic risk groups may reflect the relatively short follow-up and the limited sample size.

Elevated beta-2-microglobulin levels (>3.5 mg/L) emerged as a strong predictor of both PFS and OS. Patients with normal beta-2-microglobulin values (30 patients, 34%) experienced significantly better outcomes, suggesting a more aggressive disease biology in patients with elevated levels. Although beta-2-microglobulin may be influenced by renal function, renal impairment did not significantly affect survival outcomes in our cohort, supporting the independent prognostic value of this marker.

In the overall population, median PFS was not reached, with an estimated 40-month PFS rate of 68% (Figure 2). Outcomes were particularly favorable among patients achieving deep responses. Patients achieving at least a PR—and especially those achieving a VGPR or better—experienced prolonged PFS and OS (Figure 3). These findings reinforce the concept that depth of response remains a key determinant of long-term outcome, even in the era of monoclonal antibody-based therapies. When compared with the MAIA trial, survival outcomes in our real-world cohort appear consistent. At 40 months, the estimated OS was 74% in the overall population and 82% among patients achieving ≥VGPR. These results suggest that the benefits observed in randomized clinical trials can be reproduced in routine clinical practice, even in older and frailer patients. Treatment was generally well tolerated. Gastrointestinal toxicity and hematological adverse events were the most common treatment-related complications. Lenalidomide dose reductions were frequently required (55 patients, 57%), generally due to gastrointestinal toxicity, but did not adversely affect survival outcomes. Gastrointestinal toxicity is a common side effect of the drug and was treated with drugs to reduce intestinal motility until recovery or a significant reduction in symptoms; if toxicity persisted, lenalidomide reduction was applied. This finding supports dose optimization strategies to improve tolerability without compromising efficacy. Anemia and neutropenia were treated with growth factors and a reduction in lenalidomide was rarely required. The incidence of grade 3–4 infections, mainly pneumonia, was 15.3% (9 patients). Infections of any grade were also the main cause of treatment cycle delays; 25 patients (26%) delayed at least one cycle, 56% of which were due to infection. Corticosteroid reduction was applied in cases of grade 3–4 infections. The incidence of grade 3–4 infections was relatively low considering the advanced age of the study population, possibly reflecting the proactive use of growth factors and infection prophylaxis. Finally, while novel quadruplet regimens incorporating anti-CD38 monoclonal antibodies have demonstrated impressive efficacy, such intensive approaches may not be suitable for all TIE-NDMM patients, particularly those who are frail or very elderly. In this context, D-Rd remains a highly effective and well-tolerated frontline option for selected patients.

## 5. Conclusions

The main limitations of this study are its retrospective design and the absence of a control group. In addition, the limited availability of cytogenetic data may reduce the strength of some subgroup analyses, as may the absence of an MRD study. Furthermore, differences between the clinical characteristics of our real-world population and those of patients enrolled in clinical trials limit the feasibility of direct comparisons. Therefore, the results of this analysis should be considered hypothesis-generating.

This real-world survey confirms the efficacy and tolerability of the D-Rd combination in TIE-NDMM patients, supporting and extending the favorable outcomes reported in the MAIA trial to routine clinical practice. More than half of the patients achieved a deep response, translating into prolonged progression-free and overall survival. In particular, patients achieving at least a partial response—and especially those achieving a very good partial response or better—appear to have a high likelihood of long-term disease control without the immediate need for subsequent therapies.

Although novel quadruplet regimens offer the potential for further improvements in survival, they may not be suitable for all patients. Our findings support the frontline use of D-Rd as a safe and effective option for selected TIE-NDMM patients, particularly those who are frail or elderly and not candidates for more intensive treatment strategies.

## Figures and Tables

**Table 1:** Patients’ characteristics.

Characteristics			Patients (Total n. 96)
Sex	M-F	n. (%)	48 (50)–48 (50)
Age (y) median (range)			74 (57–88)
	Age ≤ 70	n. (%)	23 (24)
	Age ≥ 75	n. (%)	32 (33)
Frailty n. (%)			
	Not Frail		21 (22)
	Frail		75 (78)
Cytogenetic Risk n. (%) *			
	Standard		20 (21)
	High		19 (20)
	Very high		5 (5)
	del17		7 (7)
	Unknown		52 (54)
ISS Stage n. (%)			
	I		24 (25)
	II		21 (22)
	III		41 (43)
	Unknown		10 (10)
Hb g/dL median-mean			10.9–11.8
Creatinine mg/dL mean			1.1
Creatinine clearance (mL/min) mean			70
EMD n. (%)			8 (8)
Para-osseous tissue n. (%)			8 (8)
High LDH n. (%) C ^+^			21 (22)
High beta2-microglobulin n. (%) ^¶^			59 (62)
Lenalidomide reduction n. (%)			55 (57)
Responses n. (%)			
	SD		3 (3)
	MR		3 (3)
	PR		29 (30)
	VGPR		31 (32)
	CR		26 (27)
	Unknown		4 (4)
ORR n. (%)			
	≥PR		86 (90)
	≥VGPR		57 (59)
	Unknown		4 (4)

The percentages may not add up to 100 due to rounding. * Cytogenetic risk is based on the presence of del17, gain1, t(4;14), t(14;16) and t(16;20): the absence of any cytogenetic alteration is considered standard risk, the presence of one cytogenetic alteration is considered high-risk and of ≥2 a very-high risk. ^+^ High LDH is defined as patients with LDH above the upper limit of the normal range. ^¶^ High beta2-microglobulin is defined as patients with values > 3.5 mg/L. ISS, international Staging System; Hb, Hemoglobin; EMD, Extra-Medullary Disease; LDH, Lactate Dehydrogenase; SD, Stable Disease; MR, Minimal Response; PR, Partial Response; VGPR, Very Good Partial Response; CR, Complete Response; ORR, Overall Response Rate.

**Table 2:** Grade 3–4 Adverse Events (AEs).

Adverse Event	Patients (n. 96 Patients) n. (%)
**Hematological AEs**	
Anemia	13 (14)
Thrombocytopenia	2 (2)
Neutropenia	17 (18)
**Non-hematological AEs**	
Neuropathy	7 (7)
Infections	14(15)
Gastrointestinal	18 (19)
Fatigue	5 (5)

## Data Availability

The data presented in this study are available from the corresponding author upon reasonable request.

## References

[1] Greipp P.R., San Miguel J., Durie B.G., Crowley J.J., Barlogie B., Blade J., Boccadoro M., Child J.A., Avet-Loiseau H., Kyle R.A. (2005). International staging system for multiple myeloma. J. Clin. Oncol..

[2] Cote J., LeBlanc R., Mian H., Chu M.P., McCurdy A., Masih-Khan E., Su J., Jimenez-Zepeda V.H., Song K., Louzada M. (2023). Real-world results of autologous stem cell transplantation in newly diagnosed multiple myeloma: A report from the Canadian Myeloma Research Group database. Blood Cancer J..

[3] Cavo M., Tosi P., Zamagni E., Cellini C., Tacchetti P., Patriarca F., Di Raimondo F., Volpe E., Ronconi S., Cangini D. (2007). Prospective, randomized study of single compared with double autologous stem-cell transplantation for multiple myeloma: Bologna 96 clinical study. J. Clin. Oncol..

[4] Benboubker L., Dimopoulos M.A., Dispenzieri A., Catalano J., Belch A.R., Cavo M., Pinto A., Weisel K., Ludwig H., Bahlis N. (2014). Lenalidomide and dexamethasone in transplant-ineligible patients with myeloma. N. Engl. J. Med..

[5] Rajkumar S.V. (2024). Multiple myeloma: 2024 update on diagnosis, risk-stratification, and management. Am. J. Hematol..

[6] Del Fabro V., Di Giorgio M.A., Leotta V., Duminuco A., Bellofiore C., Markovic U., Romano A., Bulla A., Curto Pelle A., Elia F. (2023). Lenalidomide plus Dexamethasone Combination as First-Line Oral Therapy of Multiple Myeloma Patients: A Unicentric Real-Life Study. Cancers.

[7] Palumbo A., Anderson K. (2011). Multiple myeloma. N. Engl. J. Med..

[8] van de Donk N.W., Janmaat M.L., Mutis T., Lammerts van Bueren J.J., Ahmadi T., Sasser A.K., Lokhorst H.M., Parren P.W. (2016). Monoclonal antibodies targeting CD38 in hematological malignancies and beyond. Immunol. Rev..

[9] van de Donk N., Usmani S.Z. (2018). CD38 Antibodies in Multiple Myeloma: Mechanisms of Action and Modes of Resistance. Front. Immunol..

[10] de Weers M., Tai Y.T., van der Veer M.S., Bakker J.M., Vink T., Jacobs D.C., Oomen L.A., Peipp M., Valerius T., Slootstra J.W. (2011). Daratumumab, a novel therapeutic human CD38 monoclonal antibody, induces killing of multiple myeloma and other hematological tumors. J. Immunol..

[11] Nijhof I.S., Groen R.W., Noort W.A., van Kessel B., de Jong-Korlaar R., Bakker J., van Bueren J.J., Parren P.W., Lokhorst H.M., van de Donk N.W. (2015). Preclinical Evidence for the Therapeutic Potential of CD38-Targeted Immuno-Chemotherapy in Multiple Myeloma Patients Refractory to Lenalidomide and Bortezomib. Clin. Cancer Res..

[12] Krejcik J., Casneuf T., Nijhof I.S., Verbist B., Bald J., Plesner T., Syed K., Liu K., van de Donk N.W., Weiss B.M. (2016). Daratumumab depletes CD38+ immune regulatory cells, promotes T-cell expansion, and skews T-cell repertoire in multiple myeloma. Blood.

[13] Markovic U., Romano A., Del Fabro V., Bellofiore C., Bulla A., Parisi M.S., Leotta S., Gentile M., Cangialosi C., Vincelli I. (2021). Daratumumab as Single Agent in Relapsed/Refractory Myeloma Patients: A Retrospective Real-Life Survey. Front. Oncol..

[14] Facon T., Kumar S., Plesner T., Orlowski R.Z., Moreau P., Bahlis N., Basu S., Nahi H., Hulin C., Quach H. (2019). Daratumumab plus Lenalidomide and Dexamethasone for Untreated Myeloma. N. Engl. J. Med..

[15] Facon T., Kumar S.K., Plesner T., Orlowski R.Z., Moreau P., Bahlis N., Basu S., Nahi H., Hulin C., Quach H. (2021). Daratumumab, lenalidomide, and dexamethasone versus lenalidomide and dexamethasone alone in newly diagnosed multiple myeloma (MAIA): Overall survival results from a randomised, open-label, phase 3 trial. Lancet Oncol..

[16] Facon T., Moreau P., Weisel K., Goldschmidt H., Usmani S.Z., Chari A., Plesner T., Orlowski R.Z., Bahlis N., Basu S. (2025). Daratumumab/lenalidomide/dexamethasone in transplant-ineligible newly diagnosed myeloma: MAIA long-term outcomes. Leukemia.

[17] Moreau P., Facon T., Usmani S.Z., Bahlis N., Raje N., Plesner T., Orlowski R.Z., Basu S., Nahi H., Hulin C. (2025). Daratumumab plus lenalidomide/dexamethasone in untreated multiple myeloma: Analysis of key subgroups of the MAIA study. Leukemia.

[18] Durie B.G.M., Hoering A., Abidi M.H., Rajkumar S.V., Epstein J., Kahanic S.P., Thakuri M., Reu F., Reynolds C.M., Sexton R. (2017). Bortezomib with lenalidomide and dexamethasone versus lenalidomide and dexamethasone alone in patients with newly diagnosed myeloma without intent for immediate autologous stem-cell transplant (SWOG S0777): A randomised, open-label, phase 3 trial. Lancet.

[19] Mateos M.V., Cavo M., Blade J., Dimopoulos M.A., Suzuki K., Jakubowiak A., Knop S., Doyen C., Lucio P., Nagy Z. (2020). Overall survival with daratumumab, bortezomib, melphalan, and prednisone in newly diagnosed multiple myeloma (ALCYONE): A randomised, open-label, phase 3 trial. Lancet.

[20] Mateos M.-V., San-Miguel J., Cavo M., Suzuki K., Jakubowiak A., Knop S., Doyen C., Lucio P., Nagy Z., Pour L. (2025). Bortezomib, melphalan, and prednisone with or without daratumumab in transplant-ineligible patients with newly diagnosed multiple myeloma (ALCYONE): Final analysis of an open-label, randomised, multicentre, phase 3 trial. Lancet Oncol..

[21] Rosiñol L., Oriol A., Rios R., Sureda A., Blanchard M.J., Hernández M.T., Martínez-Martínez R., Moraleda J.M., Jarque I., Bargay J. (2019). Bortezomib, lenalidomide, and dexamethasone as induction therapy prior to autologous transplant in multiple myeloma. Blood.

[22] Facon T., San-Miguel J., Dimopoulos M.A., Mateos M.V., Cavo M., van Beekhuizen S., Yuan Z., Mendes J., Lam A., He J. (2022). Treatment Regimens for Transplant-Ineligible Patients With Newly Diagnosed Multiple Myeloma: A Systematic Literature Review and Network Meta-analysis. Adv. Ther..

[23] Voorhees P.M., Sborov D.W., Laubach J., Kaufman J.L., Reeves B., Rodriguez C., Chari A., Silbermann R., Costa L.J., Anderson L.D. (2023). Addition of daratumumab to lenalidomide, bortezomib, and dexamethasone for transplantation-eligible patients with newly diagnosed multiple myeloma (GRIFFIN): Final analysis of an open-label, randomised, phase 2 trial. Lancet Haematol..

[24] Sonneveld P., Dimopoulos M.A., Boccadoro M., Quach H., Ho P.J., Beksac M., Hulin C., Antonioli E., Leleu X., Mangiacavalli S. (2024). Daratumumab, Bortezomib, Lenalidomide, and Dexamethasone for Multiple Myeloma. N. Engl. J. Med..

[25] Moreno L., Perez C., Zabaleta A., Manrique I., Alignani D., Ajona D., Blanco L., Lasa M., Maiso P., Rodriguez I. (2019). The Mechanism of Action of the Anti-CD38 Monoclonal Antibody Isatuximab in Multiple Myeloma. Clin. Cancer Res..

[26] Facon T., Dimopoulos M.A., Leleu X.P., Beksac M., Pour L., Hajek R., Liu Z., Minarik J., Moreau P., Romejko-Jarosinska J. (2024). Isatuximab, Bortezomib, Lenalidomide, and Dexamethasone for Multiple Myeloma. N. Engl. J. Med..

[27] Leleu X., Hulin C., Lambert J., Bobin A., Perrot A., Karlin L., Roussel M., Montes L., Cherel B., Chalopin T. (2024). Isatuximab, lenalidomide, dexamethasone and bortezomib in transplant-ineligible multiple myeloma: The randomized phase 3 BENEFIT trial. Nat. Med..

[28] Ocio E.M., Perrot A., Bories P., San-Miguel J.F., Blau I.W., Karlin L., Martinez-Lopez J., Wang S.Y., Bringhen S., Marcatti M. (2023). Efficacy and safety of isatuximab plus bortezomib, lenalidomide, and dexamethasone in patients with newly diagnosed multiple myeloma ineligible/with no immediate intent for autologous stem cell transplantation. Leukemia.

[29] Manier S., Dimopoulos M.A., Leleu X.P., Moreau P., Cavo M., Goldschmidt H., Orlowski R.Z., Tron M., Tekle C., Bregeault M.F. (2025). Isatuximab plus bortezomib, lenalidomide, and dexamethasone for transplant-ineligible newly diagnosed multiple myeloma patients: A frailty subgroup analysis of the IMROZ trial. Haematologica.

[30] Dimopoulos M.A., Terpos E., Boccadoro M., Moreau P., Mateos M.V., Zweegman S., Cook G., Engelhardt M., Delforge M., Hajek R. (2025). EHA-EMN Evidence-Based Guidelines for diagnosis, treatment and follow-up of patients with multiple myeloma. Nat. Rev. Clin. Oncol..

[31] Rajkumar S.V., Dimopoulos M.A., Palumbo A., Blade J., Merlini G., Mateos M.V., Kumar S., Hillengass J., Kastritis E., Richardson P. (2014). International Myeloma Working Group updated criteria for the diagnosis of multiple myeloma. Lancet Oncol..

[32] Markovic U., Leotta V., Tibullo D., Giubbolini R., Romano A., Del Fabro V., Parrinello N.L., Cannizzaro M.T., Di Raimondo F., Conticello C. (2020). Serum free light chains and multiple myeloma: Is it time to extend their application?. Clin. Case Rep..

[33] Markovic U., Romano A., Bellofiore C., Condorelli A., Garibaldi B., Bulla A., Duminuco A., Del Fabro V., Di Raimondo F., Conticello C. (2021). Role of Serum Free Light Chain Assay in Relapsed/Refractory Multiple Myeloma. A Real-Life Unicentric Retrospective Study. Cancers.

[34] Duminuco A., Nardo A., Orofino A., Giunta G., Conticello C., Del Fabro V., Chiarenza A., Parisi M.S., Figuera A., Leotta S. (2024). Efficacy and safety of tixagevimab-cilgavimab versus SARS-CoV-2 breakthrough infection in the hematological conditions. Cancer.

[35] Duminuco A., Romano A., Leotta D., La Spina E., Cambria D., Bulla A., Del Fabro V., Tibullo D., Giallongo C., Palumbo G.A. (2023). Clinical outcome of SARS-CoV-2 infections occurring in multiple myeloma patients after vaccination and prophylaxis with tixagevimab/cilgavimab. Front. Oncol..

[36] Facon T., Dimopoulos M.A., Meuleman N., Belch A., Mohty M., Chen W.M., Kim K., Zamagni E., Rodriguez-Otero P., Renwick W. (2020). A simplified frailty scale predicts outcomes in transplant-ineligible patients with newly diagnosed multiple myeloma treated in the FIRST (MM-020) trial. Leukemia.

[37] Hillengass J., Usmani S., Rajkumar S.V., Durie B.G.M., Mateos M.V., Lonial S., Joao C., Anderson K.C., Garcia-Sanz R., Riva E. (2019). International myeloma working group consensus recommendations on imaging in monoclonal plasma cell disorders. Lancet Oncol..

[38] Diaconescu D., Soare D.S., Marinescu C.E., Ene G.E., Bumbea H. (2025). Beyond the bone marrow: A review of therapeutic approaches for extramedullary disease in multiple myeloma and the significance of MRD assessment. J. Med. Life.

[39] Talarico M., Barbato S., Cattabriga A., Sacchetti I., Manzato E., Restuccia R., Masci S., Bigi F., Puppi M., Iezza M. (2025). Diagnostic Innovations: Advances in imaging techniques for diagnosis and follow-up of multiple myeloma. J. Bone Oncol..

[40] Cani L., Petrucci M.T., Mancuso K., Zambello R., Paris L., Aquino S., Lotti F., Vassallo F., Pescosta N., Quaresima M. (2025). Clinicians’ Perspectives and Methodological Application of Fluorescence in situ Hybridization (FISH) to Define Cytogenetic Risk in Multiple Myeloma: An Italian, Real-World, Survey-Based Report From the European Myeloma Network (EMN) Italy. Clin. Lymphoma Myeloma Leuk..

[41] D’Agostino M., Cairns D.A., Lahuerta J.J., Wester R., Bertsch U., Waage A., Zamagni E., Mateos M.V., Dall’Olio D., van de Donk N. (2022). Second Revision of the International Staging System (R2-ISS) for Overall Survival in Multiple Myeloma: A European Myeloma Network (EMN) Report Within the HARMONY Project. J. Clin. Oncol..

